# Age-related decline in cognitive flexibility is associated with the levels of hippocampal neurogenesis

**DOI:** 10.3389/fnins.2023.1232670

**Published:** 2023-08-14

**Authors:** Evgeny M. Amelchenko, Dmitri V. Bezriadnov, Olga A. Chekhov, Konstantin V. Anokhin, Alexander A. Lazutkin, Grigori Enikolopov

**Affiliations:** ^1^Center for Developmental Genetics, Stony Brook, NY, United States; ^2^Department of Anesthesiology, Stony Brook University, Stony Brook, NY, United States; ^3^P.K. Anokhin Research Institute of Normal Physiology RAS, Moscow, Russia; ^4^Institute for Advanced Brain Studies, Lomonosov Moscow State University, Moscow, Russia; ^5^Institute of Higher Nervous Activity and Neurophysiology RAS, Moscow, Russia

**Keywords:** adult-born neurons, aging, cognitive flexibility, hippocampal neurogenesis, neuronal maturation, search strategies, spatial learning

## Abstract

Aging is associated with impairments in learning, memory, and cognitive flexibility, as well as a gradual decline in hippocampal neurogenesis. We investigated the performance of 6-and 14-month-old mice (considered mature adult and late middle age, respectively) in learning and memory tasks based on the Morris water maze (MWM) and determined their levels of preceding and current neurogenesis. While both age groups successfully performed in the spatial version of MWM (sMWM), the older mice were less efficient compared to the younger mice when presented with modified versions of the MWM that required a reassessment of the previously acquired experience. This was detected in the reversal version of MWM (rMWM) and was particularly evident in the context discrimination MWM (cdMWM), a novel task that required integrating various distal cues, local cues, and altered contexts and adjusting previously used search strategies. Older mice were impaired in several metrics that characterize rMWM and cdMWM, however, they showed improvement and narrowed the performance gap with the younger mice after additional training. Furthermore, we analyzed the adult-born mature and immature neurons in the hippocampal dentate gyrus and found a significant correlation between neurogenesis levels in individual mice and their performance in the tasks demanding cognitive flexibility. These results provide a detailed description of the age-related changes in learning and memory and underscore the importance of hippocampal neurogenesis in supporting cognitive flexibility.

## Introduction

In humans and animals, aging is associated with a gradual impairment of learning, memory, and executive function (Salthouse, 2009; Samson and Barnes, 2013; Nyberg and Pudas, 2019; McQuail et al., 2020). Aging is also associated with a decrease in cognitive (behavioral) flexibility – the ability to switch between mental tasks, adapt to new environments, and adjust strategies in response to changing circumstances. Cognitive flexibility is a critical component of the executive function and deficits in cognitive flexibility may contribute to age-related cognitive decline.

Not all cognitive functions show deterioration with age, as certain aspects such as procedural, automatic, and verbal memory tend to be preserved during normal aging (Nyberg and Pudas, 2019; McQuail et al., 2020). Furthermore, the decline in certain cognitive functions can be partially mitigated by increased learning and cognitive training. While a wide range of specialized tests can detect subtle age-related changes in cognitive performance in humans, there is a limited repertoire of such tests available for animal models, such as rodents. As a result, certain nuanced features affected by aging may go unnoticed due to the lower resolution of behavioral tasks designed for animals.

Another hallmark of aging is the decrease in production of new neurons in the dentate gyrus (DG) of the hippocampus, a brain region that supports neurogenesis in humans and animals long after birth (Kuhn et al., 1996; Encinas et al., 2011; Boldrini et al., 2018; Kempermann et al., 2018; Pilz et al., 2018; Shetty et al., 2018; Sorrells et al., 2018; Lazutkin et al., 2019; Tobin et al., 2019; Toda et al., 2019; Urban et al., 2019; Bottes et al., 2021; Ibrayeva et al., 2021; Wu et al., 2023). Adult-born hippocampal neurons are believed to play diverse roles, including their involvement in distinguishing subtle differences in familiar contexts (pattern separation), supporting behavioral flexibility, and promoting active forgetting (Saxe et al., 2006; Clelland et al., 2009; Arruda-Carvalho et al., 2011; Sahay et al., 2011b; Burghardt et al., 2012; Niibori et al., 2012; Tronel et al., 2012; Aimone et al., 2014; Akers et al., 2014; Epp et al., 2016; McAvoy et al., 2016; Anacker and Hen, 2017; Toda et al., 2019; Yu et al., 2019; Lods et al., 2021; Koehl et al., 2022). A growing body of evidence indicates that decreased hippocampal neurogenesis is associated with impaired performance in a variety of learning and memory tasks [e.g., MWM and contextual fear conditioning (Hernandez-Mercado and Zepeda, 2021)]; moreover, experimental enhancement of neurogenesis has been shown to improve performance in relevant cognitive tests (Sahay et al., 2011a; McAvoy and Sahay, 2017; Berdugo-Vega et al., 2020; Montaron et al., 2020).

In this study we aimed to investigate whether the effects of aging can be detected using a novel set of tasks that we developed to assess changes in cognitive flexibility in mice (Amelchenko et al., 2023). We show that aging leads to the adoption of inefficient and spatially imprecise search strategies, resulting in impaired learning; however, this deficiency can be overcome through an extended training period. Furthermore, we sought to characterize the decline in production of neural stem and progenitor cells that accompanies aging. We found a pronounced correlation between the levels of immature and mature neurons in the dentate gyri of individual mice and their performance in tasks that require cognitive flexibility. Thus, our study reveals age-dependent impairment of several features that characterize learning, memory, and cognitive flexibility in mice and provides evidence for a correlation between adult hippocampal neurogenesis and cognitive flexibility.

## Materials and methods

### Mice

Adult male Nestin-CFPnuc mice (Mignone et al., 2004; Encinas et al., 2006; Enikolopov et al., 2015) maintained on C57BL/6J background, were used for all experiments. Prior and during the experiment, mice were housed in groups of 2–4 animals per cage under standard conditions with 12/12 h light–dark cycle, in cages 36х21х13.5 cm, with food and water available *ad libitum*. All experiments were conducted in compliance with the requirements, regulations, and guidelines issued by the National Institutes of Health and Stony Brook University.

We examined mice of different ages, dividing them into two groups. The first group consisted of mice that were 6 months old at the start of the behavioral training sessions (referred to as the 6MO group, *n* = 13). The second group consisted of mice that were 14 months old at the start of the behavioral training sessions (referred to as the 14MO group, *n* = 15). The same animals from the 6MO and 14MO groups were used for all experiments.

### Cell labeling

To label dividing cells in the brain, experimental animals were injected with 5-ethynyl-2′-deoxyuridine (EdU, 123 mg/kg, Invitrogen, USA) intraperitoneally (Podgorny et al., 2018; Ivanova et al., 2022) 6 weeks before behavioral training. After the injection, all mice were returned to their home cages and housed under conventional conditions before the beginning of behavioral experiments.

### Morris water maze training

**Figure 1:**
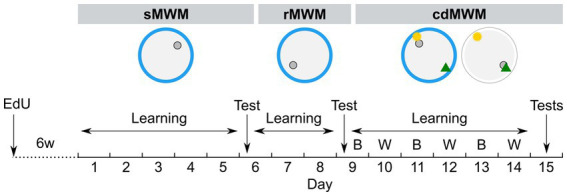
Experimental timeline. Mice were trained in succession of three tasks: spatial MWM (sMWM) learning – days 1–5, sMWM memory test – day 6; reversal MWM (rMWM) learning – days 6–8, rMWM memory test – day 9; context discrimination (cdMWM) learning in daily alternating blue (B) and white (W) pools with a pair of local cues, that context-dependently signaled platform location – days 9–14, cdMWM memory tests in the blue and white pools – day 15. Blue ring represents the blue swimming pool, white ring represents the white swimming pool, small grey circle represents platform location, note different platform location in different tasks; yellow star and green triangle represents local cues introduced for training in the cdMWM task.

Mice were transferred from the animal facility to the experimental room 1 h prior to the beginning of the experimental procedures. The mice were subjected to three consecutive Morris water maze- (MWM)-based tasks: spatial MWM (sMWM), followed by reversal MWM (rMWM), which was followed by context discrimination MWM (cdMWM). For the training in the sMWM (Figure 1), animals were presented with a circular pool (120 cm in diameter) made of blue plastic (Noldus, the Netherlands). The water was made opaque with non-toxic white paint and maintained at 23–24°C. Several distal cues were affixed to the walls surrounding the pool. Each mouse performed five 60 s trials per day, with 30 min inter-trial intervals, for five consecutive days, with the objective to locate a submerged platform positioned 0.5 cm below the water surface in one of the virtual quadrants (the target quadrant, T). At the beginning of each trial, a mouse was released into the pool from one of three virtual quadrants that did not contain the platform. If a mouse failed to find the platform, the experimenter gently guided it to the platform. The mouse was allowed to remain on the platform for 30 s. The platform position remained constant throughout the five training days. On day 6, the mice underwent a spatial memory test involving swimming for 60 s in the pool without the platform.

Training in rMWM (Figure 1) started 2 h after the sMWM memory test. The hidden platform was reintroduced to the pool but was placed in the quadrant opposite to that used during sMWM training. The distal cues on the walls remained the same and were located at the same positions relative to the pool. The mice were given three additional days to learn the new platform location. The overall procedure was identical to that described for sMWM. On day 9, the mice were subjected to a 60 s memory test without the platform.

cdMWM training (Figure 1) began 2 h after the rMWM memory test (day 9). The same blue plastic pool and the same distal cues on the walls were used. The platform was relocated to a new quadrant and two local cues (beacons) – a yellow rubber ball and a multicolor pyramid – were suspended 20 cm above the water surface. One beacon was placed above the platform (the goal cue), while the other was positioned above the opposite quadrant (the false cue). On the following day (day 10), each mouse was placed in a pool identical to the blue pool except it was constructed from white plastic. This pool was located in the adjacent room with different distal cues on the walls. Two beacons identical to those used in the blue pool (a ball and a pyramid) were suspended above the pool, but the goal cue and the false cue were switched, i.e., if the ball indicated the platform location in the blue pool, it served as the false cue in the white pool, while the pyramid became the new goal cue. The assignment of beacons indicating the platform location was counterbalanced between the pools: for half of the animals in each group in a particular pool the goal cue was the ball, whereas for the other half in the same pool the goal cue was the pyramid. Therefore, to choose the correct local cue indicating the platform location, each mouse needed to discriminate among the contextual factors of the room, pool color, and distal cues. Each mouse underwent five daily sessions lasting 60 s each, and after reaching the platform was allowed an additional 30 s on the platform. The pools were alternated daily for six days (day 9 through day 14). On day 15, the mice underwent 60 s memory tests in the blue and white pools, separated by a 2 h interval. The timeline of the tasks is depicted in Figure 1.

### Behavioral parameters and analysis

Animals’ behavior was recorded with the video camera mounted above the pool. For behavioral tracking, EthoVision XT, versions 8 and 17 (Noldus, the Netherlands) was used, and the following behavioral parameters were extracted from the video: *escape latency* – time to reach the platform in training trial; *fraction of time spent in each quadrant* (in %) in the memory test. For the classification analysis of the search strategies, we used the Pathfinder software (Cooke et al., 2019). Raw tracking files with *xy coordinates over time* were extracted from the EthoVision XT and uploaded to the Pathfinder. The following parameters, empirically determined for our combination of the pool and platform size and location were used: Goal Position (x,y): varied between sMWM, rMWM, cdMWM; Goal Diameter (cm): 10; Maze Diameter (cm): 120; Maze Centre (x,y): 0,0; Angular Corridor Width (degrees): 20; Chaining Angular Width (cm): 15; Thigmotaxis Zone Size (cm): 15. A trial was truncated when the animal reached goal location. As a result, each track was assigned to one of the seven search strategies: direct path, directed search, focal search, indirect search, scanning, random search, thigmotaxis (from the most spatially precise and efficient to the least one). In wild-type healthy rodents, learning leads to a decrease of the fraction of inefficient, spatially imprecise search strategies (presumably, hippocampal-independent) and is accompanied by an increase of the fraction of more efficient, spatially specific, and, presumably, hippocampal-dependent, strategies (indirect search, focal search, directed search, direct path) (Garthe et al., 2009, 2014, 2016; Cooke et al., 2019). The repertoire of strategies used in every trial was presented as a percentage of each strategy used by animals for each group. *The ideal path error* (IPE) extracted from the Pathfinder output file was calculated as follows: *the summed error of the search path (cm) = the cumulative actual path distance (cm) – the cumulative ideal path (cm)*. This parameter served as another metric to characterize effectiveness of search strategies: the IPE is expected to be lower if mostly spatially precise strategies were used, and higher if mostly spatially imprecise strategies were used. *The preference score* was calculated as the time spent in the target (T) quadrant divided by the cumulative time spent in both the T quadrant and the opposite (O) quadrant: *(time in T) × 100/(time in T + time in O)* was used to directly compare the quadrant discrimination between the experimental groups*. The correct first choice (CFC)* score was calculated by subtracting the latency to reach the goal cue from the latency to reach the false cue.

### Immunohistochemistry

After the completion of behavioral testing, mice were deeply anesthetized with a mixture of Zoletil 100 (40 mg/kg) and xylazine (5 mg/kg) and transcardially perfused with 30 mL of ice-cold PBS followed by 30 mL of 4% paraformaldehyde (PFA) in phosphate buffer (PBS), pH 7.4. Brains were removed from the skull and postfixed in 4% PFA overnight at 4°C. The following day, the brains were transferred into PBS and stored until sectioning. Free-floating 50 μm-thick sagittal sections were obtained using a Leica VT1000S vibratome (Leica, Germany). The sections were collected in PBS and kept in PBS at 4°C or in cryoprotectant (1 volume of ethylene glycol, 1 volume of glycerin, and 2 volumes of PBS) at −20°C until staining. For permeabilization and blocking, sections were incubated in a solution containing 2% Triton-X100 in PBS (2% TBS) and 5% normal goat serum (Abcam, USA, ab7481) for 1 h at room temperature on a rocking platform. Next, the sections were incubated with primary antibodies in 0.2% TBS and 3% normal goat serum overnight at room temperature on a rocking platform. After washing three times in 0.2% TBS, sections were incubated with secondary antibodies in 0.2% TBS and 3% normal goat serum for 2 h at room temperature in darkness on a rocking platform. The following antibodies were used: guinea pig anti-DCX (1:2000, Millipore, USA, AB2253) and goat anti-guinea pig AlexaFluor 647 (1:500, Molecular Probes, USA, A21450); mouse anti-NeuN (1:1000, Millipore AB377) and goat anti-mouse AlexaFluor 488 (1:400, Molecular Probes, A32723). After three washings in 0.2% TBS, the click reaction was performed with AlexaFluor 555 Azide, triethylammonium salt (Invitrogen, USA, A20012) according to Salic and Mitchison (2008) and Ivanova et al. (2022). After three washes in 0.2% TBS and three washes in PBS, the sections were glass-mounted using Fluorescent Mounting Medium (DAKO, USA, S3023). The glass slides were dried horizontally overnight at room temperature in darkness, then stored at 4°C until imaging.

### Imaging and cell counting

Cell counting was performed by means of design-based stereology (Encinas and Enikolopov, 2008). One brain hemisphere was randomly selected for each animal. The hemisphere was sagittally sectioned in the lateral-to-medial direction, from the beginning of the lateral ventricle to the midline, thus covering the entire DG region. The sections were 50 μm thick and were collected in six parallel sets; thus, each set was comprised of sections that were 300 μm apart from each other in the brain. One set of 8–9 sections on average, covering the DG, was used for cell counting. The sections were imaged using a spinning-disc confocal microscope (Andor Revolution WD, Oxford Instruments, UK) with the iQ 3.1 software (Oxford Instruments, UK) and a 20x NA 0.75 objective (Nikon, Japan). All images were imported into Imaris software (v.7.6.4, Bitplane, UK) and cells were counted manually by an experimenter who was blinded to the group assignment. The cell counts for the section set were averaged, normalized to the average number of sections from all animals, and then multiplied by 6 and by 2 (the number of wells and hemispheres, respectively) to represent the total number of cells per two hippocampi.

To control the validity of cell counts, we compared the impact of between-sample variability of cell counts (i.e., the variance of cell counts within a set of brain sections from the same animal) with the impact of between-animals variability. We calculated the mean coefficient of error (CE) for the mice and compared it to the group variance (CV) (Slomianka and West, 2005; Basler et al., 2017; Slomianka, 2021). A $\frac{\mathrm{mean}\;CE_{sampling}^{2}}{CV_{group}^{2}}$ ratio less than 0.5 would indicate that between-sample variability in cell counts contributed less than 50% to the overall variability between the animals, a value considered to be acceptable as a measure of the results’ validity in most cases (Slomianka, 2021).

### Statistical analysis

Statistical analysis was performed using Prism (version 6.04, GraphPad Software, USA) and SPSS (version 28.0.0, IBM, USA). We used unpaired (independent samples, repeated measures) design for the experiments. For the analysis of escape latencies we used two-way repeated measures ANOVA followed by multiple comparisons with Sidak’s correction, with a family-wise significance level set to 0.05 (*α* = 0.05). For the analysis of the effect of age on the search strategies, we used Generalized Linear Mixed Model (GLMM). The response variable was the strategy used by an animal to reach platform in each trial. If a spatially precise strategy (direct path, directed search, focal search, indirect search) was used, it was scored “1”; if a spatially imprecise strategy (scanning, random search, thigmotaxis) was used, it was scored “0.” Age and the day of training were the predictor variables; subjects (individual animals) were added to model a random effect. A separate GLMM analysis was conducted for each of the MWM tasks. The results were presented as odds ratio (OR) of using precise search strategies over imprecise search strategies in the aged (14MO) group compared to the mature adult (6MO) group; a significant difference for the between-group pairwise comparison was determined with Bonferroni correction. The ideal path error (IPE) data were analyzed with two-way repeated measures ANOVA followed by multiple comparisons with Sidak’s correction.

To assess the distribution of time spent in quadrants in the test trials for uniformity we applied the Dirichlet distribution, using the “Dirichlet package” (Maugard et al., 2019). If the distribution was found to diverge from a uniform distribution, we performed *post-hoc* single sample *t*-tests with Bonferroni correction to compare the percentage of time spent in a particular quadrant with a theoretical value of 25%. The average distributions of the track points (mouse locations) in the test trials were represented as heatmaps generated in Ethovision XT. For the preference score (transformed with probit function from non-normally distributed data to normally distributed) and the CFC score analysis, we used Mann–Whitney *t*-test.

For the cell counts, we used Mann–Whitney *t*-test, with a significance level set to 0.05 (*α* = 0.05). A total of 10 mice from the 6MO group and 14 mice from the 14MO group were examined. In the 6MO group, one animal had pronounced hydrocephaly and was excluded from the analysis. The brain sections from two other animals showed unexpectedly high number of DCX^+^ cells [three-fold higher than in the other 24 animals; values identified as outliers using the ROUT method (Motulsky and Brown, 2006)] and were excluded from the further cell count analysis. In the 14MO group, sections from one animal were damaged during staining and processing and could not be included in the cell number analysis. Of note, these four mice did not differ significantly from the remaining mice in each of the behavioral tests.

To assess potential joint variation of individual behavioral parameters and neurogenesis, we performed correlation analysis. We used (a) escape latencies (averaged across five training trials) or cumulative number of precise search strategies used by individual mice on specific days of training (ranging from 0 – if a mouse did not use any of precise search strategies in each of five training trials, to 5 – if a mouse used only precise search strategies in each of five training trials), and (b) cell counts (DCX^+^ and EdU^+^NeuN^+^DCX^-^ cells per DG) for the same animals. We used Spearman rank correlation (following D’Agostino & Pearson omnibus normality test), with a significance level set to 0.05 (*α* = 0.05).

Graphs were plotted in Prism, the “Dirichlet package,” and Ethovision XT. Images were obtained in Imaris. Final figures were prepared in Inkscape 1.1.

## Results

### Experimental design

To investigate the effects of aging on learning, memory, behavioral flexibility, and neurogenesis, we focused on two age groups: mice that are considered mature adult (6 months old, the 6MO group) and those considered to be of late middle age (14 months old, the 14MO group), i.e., on the ages before the overt manifestation of impairments characteristic of old age (Flurkey et al., 2007). We assessed the animals’ performance in a series of consecutive MWM tasks designed to evaluate various behavioral domains and analyzed several parameters to quantify the age-related changes (scheme in Figure 1). Additionally, we tagged dividing cells using the thymidine analog EdU, administered 6 weeks prior to the start of the MWM series.

**Figure 2:**
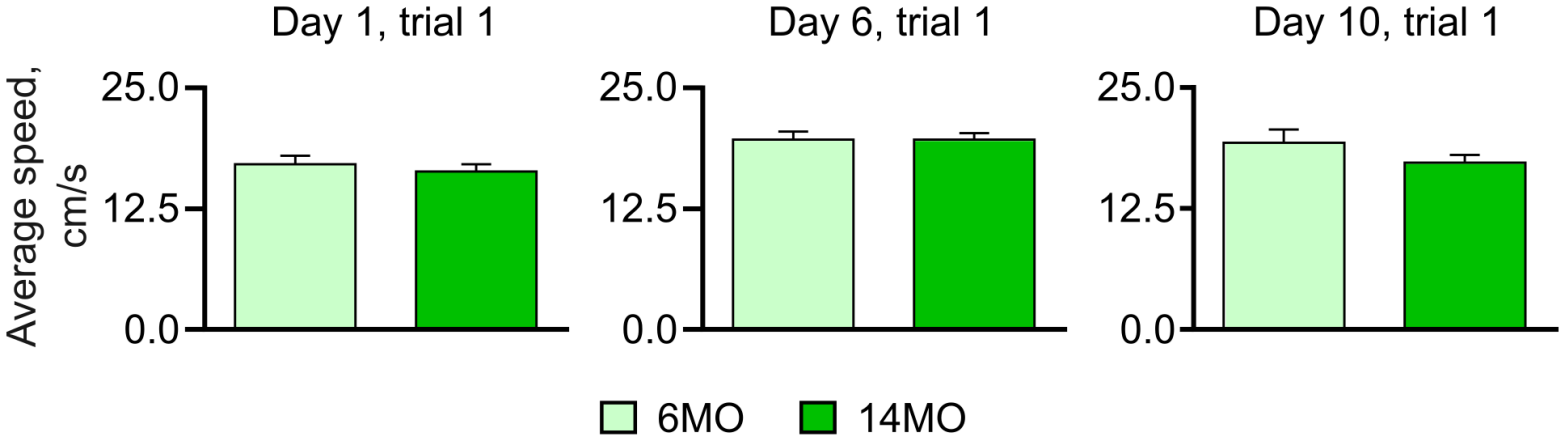
Average swim speed measured in course of training; no significant difference between the groups was found (Mann–Whitney *t*-test), data presented as Mean ± SEM.

Both the 6MO and 14MO groups were first trained for 5 days to locate a hidden escape platform in the standard spatial MWM (sMWM) task (Figure 1). On day 6, the platform was removed, and a memory test was conducted. Next, the same animals underwent 3 days of training (days 6–8) in the reversal MWM (rMWM) task where the platform was moved to the quadrant opposite to that used in the sMWM task. Following another memory test without the platform on day 9, the mice underwent training for 6 days (days 9–14) in the context discrimination MWM (cdMWM), with the color of the pool (blue and white) and the position of the beacons (a ball and a pyramid) alternating daily. The training in cdMWM was followed by memory tests in both pool configurations, with the platform removed. To evaluate the overall fitness of the animals for the task series, we measured an average swimming speed on days 1, 6, and 10 of training and found no significant differences between the 6MO and 14MO groups, indicating comparable locomotor activity of the mice of both ages (Figure 2).

### Learning, search strategies, and memory in the sMWM, rMWM, and cdMWM tasks in 6- and 14-month-old mice

#### sMWM

**Figure 3:**
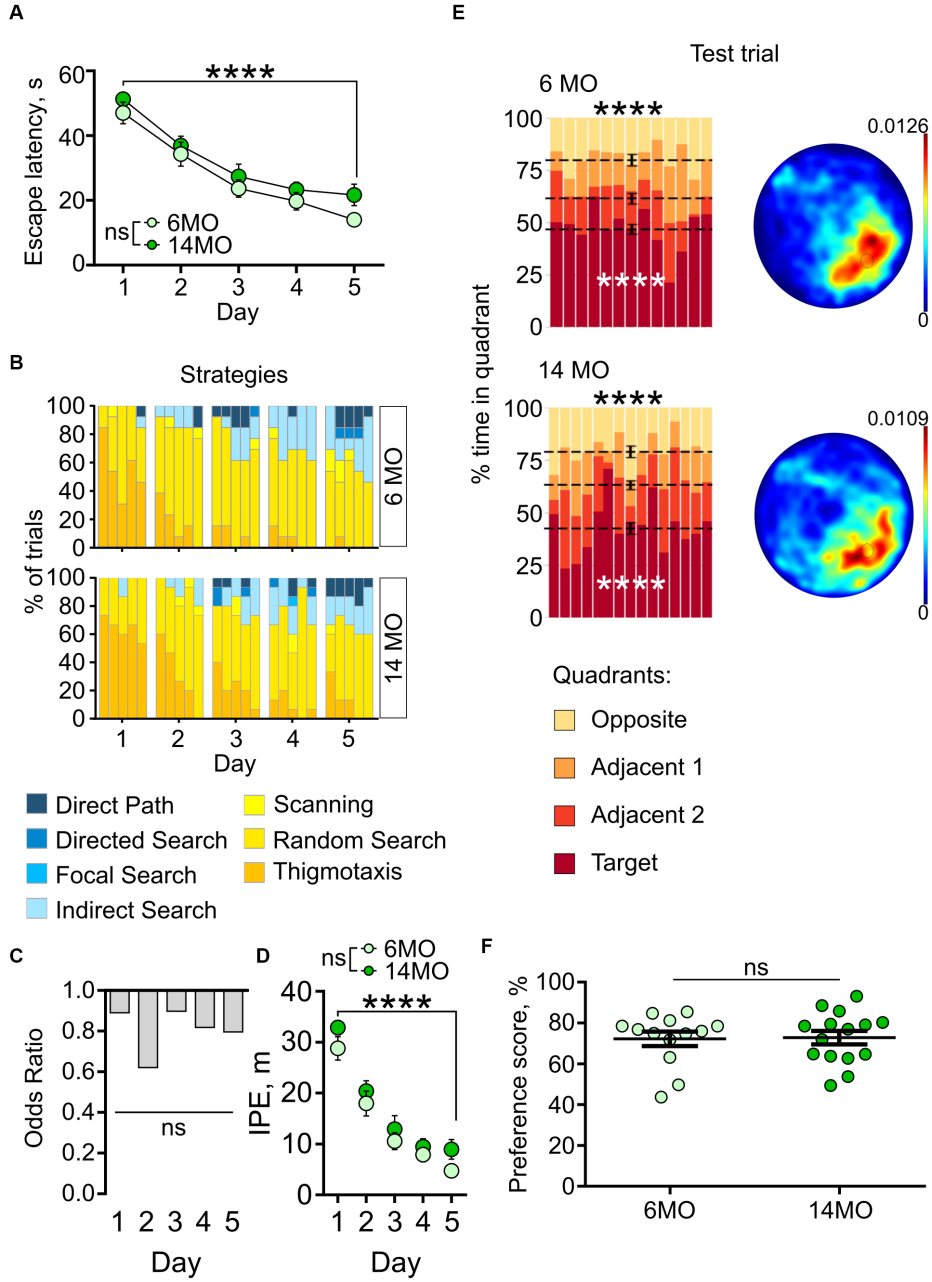
Behavior in spatial MWM (sMWM) of 6-month-old (6MO, *n* = 13) and 14-month-old (14MO, *n* = 15) mice. **(A)** Escape latencies (time to reach a hidden platform) during training; ****p < 0.0001 for each of two groups, ns – *p* > 0.05 (two-way ANOVA, multiple comparisons with Sidak’s correction), data presented as Mean ± SEM; **(B)** Search strategies in sMWM; each block of stacked bars indicates strategies used for the 5 trials for each day; **(C)** Odds ratio of spatially precise strategies use in 14MO compared with the 6MO in sMWM; ns – *p* > 0.05 (*p* values determined by fitting a generalized linear mixed effect model with binomial distribution); **(D)** Ideal path error (IPE) in sMWM; ****p < 0.0001 for each of two groups, ns – *p* > 0.05 for effect of age (two-way ANOVA, multiple comparisons with Sidak’s correction), data presented as Mean ± SEM; **(E)** Time spent in quadrants (in %) during test following sMWM training; each column represents an animal and each color represents the percent of time spent in each quadrant. Mean value for the fraction of time spent in each quadrant is represented by a black dash line and the error bar on the mean is approximated with the inverse Fisher information, ****p < 0.0001, in black symbols the results of Dirichlet distribution analysis are shown, in white symbols the results of *post-hoc* single sample *t*-test comparison (with Bonferroni correction) with the theoretical value 25% are shown; heatmaps represent average distribution of the track points (mouse locations) in the pool during test trial; **(F)** Preference score, relative time spent in T quadrant in test, dots represent individual values, ns – *p* > 0.05, data presented as Mean ± SEM.

Both groups of mice successfully learned the platform location, as evidenced by a significant reduction in escape latencies throughout the training period (Figure 3A). Two-way repeated measures ANOVA indicated a significant effect of the training day (*p* < 0.0001), but not of the animals’ age or the training day × age interaction (*p* > 0.05 for both). *Post-hoc* comparison using Sidak’s correction revealed a decrease in escape latencies from day 1 to day 5 of training in both the 6MO group (*p* < 0.0001) and the 14MO group (*p* < 0.0001), indicating improved performance in the sMWM task for both age groups.

We further analyzed the navigation strategies used by the mice during the search for the escape platform. For each trial, the overall search path was assigned to one of seven distinct search strategies (Cooke et al., 2019), ranging from inefficient spatially imprecise (e.g., thigmotaxis and random search), to highly efficient spatially precise (e.g., directed path, directed search and focal search). Over the 5 days of sMWM training, both groups showed an increase in the proportion of spatially precise search strategies and a decrease in spatially imprecise strategies (Figure 3B).

As another approach to characterize the use of different search strategies by the 6MO and 14MO groups, we applied Generalized Linear Mixed Model (GLMM) analysis to determine the relationship between the outcome variable (search strategy) and predictor variables (age and day of training). The GLMM did not reveal a significant effect of the training day × age interaction (*p* = 0.990) or of age alone (*p* = 0.895), but it did indicate a significant effect of training day (*p* < 0.001) (Figure 3C). Thus, in the sMWM task, the age of the animals did not affect the odds of using spatially precise search strategies compared to spatially imprecise strategies.

As yet another measure of the efficiency of the platform search, we determined the ideal path error (IPE), which represents the difference between the ideal path (a straight line from the release site to the platform) and the actual path taken by the mouse during the search. Both groups showed a significant decrease in the IPE from day 1 to day 5 of the sMWM task (effect of training day: *p* < 0.0001), with no effect of age (*p* = 0.1269), or the training day × age interaction (*p* = 0.8977) (Figure 3D). This indicated improved performance during learning, without significant difference in IPE between the 6MO and 14MO groups.

We next assessed the performance of the mice in the memory test by examining whether the time spent searching the platform was uniformly distributed between the quadrants. Using the Dirichlet distribution to account for the constant-sum constraints of the MWM test (Maugard et al., 2019), we found that in both age groups the distribution of time spent in the four quadrants significantly deviated from a uniform distribution (6MO: *p* < 0.0001; 14MO: *p* < 0.0001, Bonferroni correction applied here and later) (Figure 3E). *Post-hoc* single sample *t*-tests revealed that the mice spent more time in the target (T) quadrant than would be expected by chance (25%) (6MO: *p* < 0.0001; 14MO: *p* < 0.0001). Heatmaps depicting the average spatial distributions of the track points (i.e., mouse locations in the pool during the sessions) further confirmed that mice from both age groups localized their search to the general area of the platform (Figure 3E). Lastly, to compare the ability of the two groups to discriminate the quadrants, we calculated quadrant preference scores and found no overall difference between the 6MO and 14MO groups (Mann–Whitney *t*-test) (Figure 3F). Thus, each of the applied tests and metrics indicates that spatial learning and memory in the sMWM task were not impaired in the older mice compared to the younger mice.

#### rMWM

**Figure 4:**
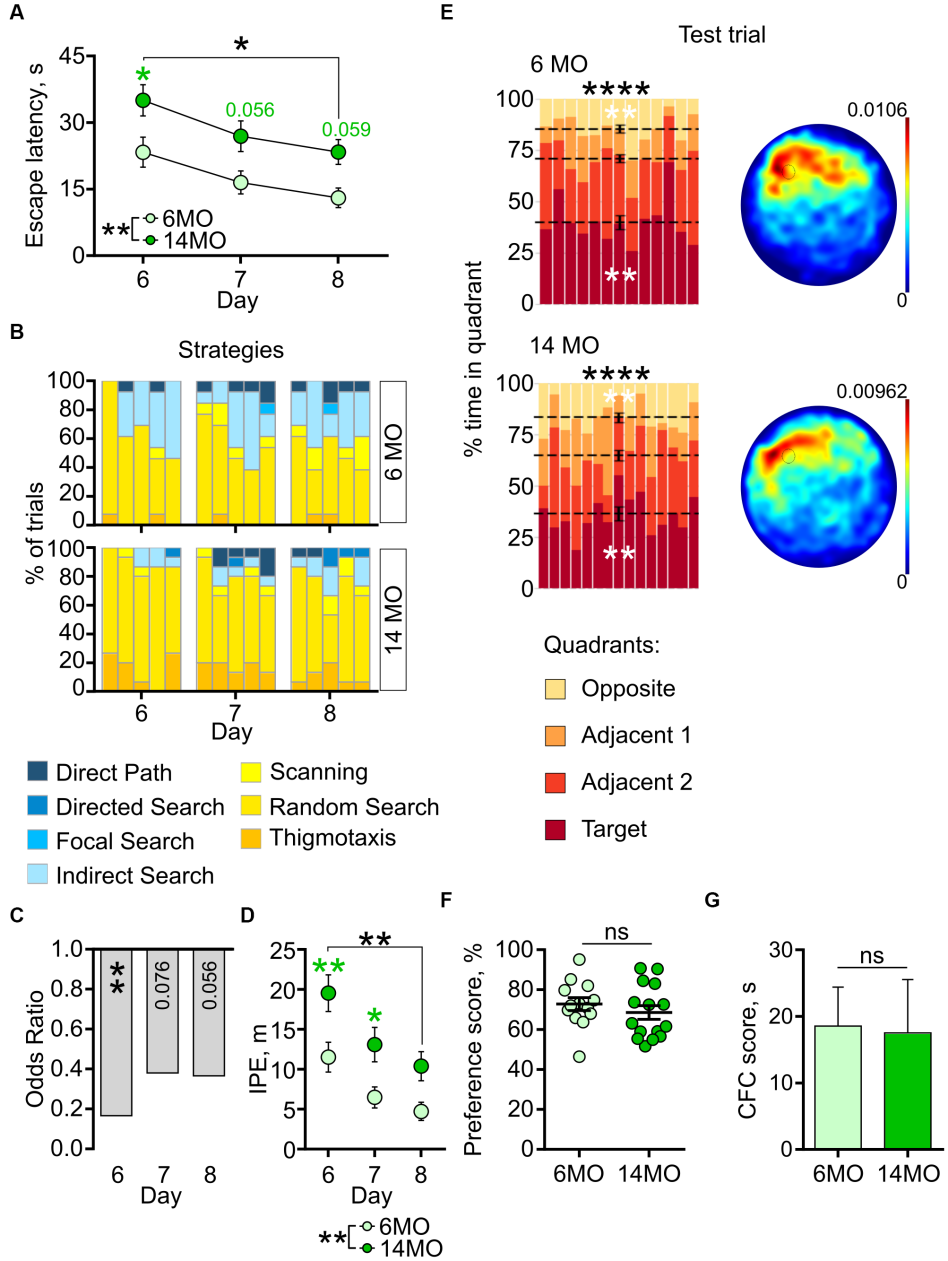
Behavior in reversal MWM (rMWM) of 6-month-old (6MO, *n* = 13) and 14-month-old (14MO, *n* = 15) mice. **(A)** Escape latencies (time to reach a hidden platform) during training; black asterisks: *p < 0.05 for day 6 vs day 8 for each of two groups, **p < 0.01 effect of age; green symbols: 14MO vs 6MO, *p < 0.05 (two-way ANOVA, multiple comparisons with Sidak’s correction), data presented as Mean ± SEM; **(B)** Search strategies in rMWM; each block of stacked bars indicates strategies used for the 5 trials for each training day; **(C)** Odds ratio of spatially precise strategies use in 14MO compared with the 6MO in rMWM; **p < 0.01 (*p* values determined by fitting a generalized linear mixed effect model with binomial distribution); **(D)** Ideal path error (IPE) in rMWM; black asterisks: both groups or between-group comparison **p < 0.01 for day 6 vs day 8 for each of two groups, **p < 0.01 effect of age; green asterisks: 14MO vs 6MO, *p < 0.05, **p < 0.01 (two-way ANOVA, multiple comparisons with Sidak’s correction), data presented as Mean ± SEM; **(E)** Time spent in quadrants (in %) during test following sMWM training; each column represents an animal and each color represents the percent of time spent in each quadrant. Mean value for the fraction of time spent in each quadrant is represented by a black dash line and the error bar on the mean is approximated with the inverse Fisher information, **p < 0.01, ****p < 0.0001, in black symbols the results of Dirichlet distribution analysis are shown, in white symbols the results of *post-hoc* single sample *t*-test comparison (with Bonferroni correction) with the theoretical value 25% are shown; heatmaps represent average distribution of the track points (mouse locations) in the pool during test trial; **(F)** Preference score, relative time spent in T quadrant in test, dots represent individual values, ns – *p* > 0.05, data presented as Mean ± SEM; **(G)** Correct first choice (CFC) score (latency to reach the false cue – latency to reach goal cue); ns – *p* > 0.05, data presented as Mean ± SEM.

We next assessed the re-learning ability of the mice by presenting them with the rMWM task. A two-way repeated measures ANOVA of the escape latencies revealed significant effects of the training day (*p* < 0.0001) and of the animals’ age (*p* = 0.0033); it did not indicate a significant interaction between the training days and age (*p* > 0.05). *Post-hoc* comparison with Sidak’s correction showed a decrease in escape latencies from day 6 to day 8 of training in both the 6MO and 14MO groups (day 8 vs day 6: *p* = 0.0104 for 6MO and *p* = 0.0016 for 14MO) (Figure 4A), indicating improved performance in the rMWM task for both age groups. Furthermore, the between-groups comparisons showed that the 14MO group had longer escape latency than the 6MO group on day 6 (*p* = 0.0263), with a trend toward longer latencies on days 7 and 8 (*p* = 0.056 and *p* = 0.059, respectively) (Figure 4A). These findings suggest that while both the 6MO and 14MO mice were capable of learning the new platform location, the 14MO mice exhibited significant impairment compared to the 6MO mice.

The impaired learning of the 14MO group was further corroborated by the analysis of search strategies. Initially, both groups predominantly utilized spatially imprecise strategies (Figure 4B, day 6 trial 1). However, starting from trial 2 on day 6 and continuing on days 7 and 8, the 6MO mice demonstrated a higher reliance on spatially precise strategies compared to the 14MO mice (Figure 4B).

The GLMM analysis revealed a significant effect of age (*p* = 0.003), but no effect of the training day or the training day × age interaction (*p* = 0.100 and *p* = 0.388, respectively) (Figure 4C). The odds ratio (OR) of using spatially precise over spatially imprecise strategies in the 14MO group compared to the 6MO group were 0.166 (*p* = 0.005) on day 6, 0.381 (*p* = 0.076) on day 7, and 0.366 (*p* = 0.056) on day 8.

For the IPE, a two-way repeated measures ANOVA revealed a significant effect of the training day (*p* = 0.0001) and age (*p* = 0039), but not of the day × age interaction (*p* = 0.0685) (Figure 4D). Further *post-hoc* analysis revealed a significantly higher IPE in the 14MO group than in the 6MO group on days 6 and 7 (*p* = 0.0098 and 0.0427, respectively). Together, these metrics indicate a pronounced learning deficit in the 14MO mice compared to the 6MO mice.

In the memory test, the distribution of time spent in the four quadrants significantly deviated from a uniform distribution for both groups (6MO: *p* < 0.0001; 14MO: *p* < 0.0001) (Figure 4E). *Post-hoc* single sample *t*-tests demonstrated that in each group mice spent more time in the T quadrant and less time in the opposite (O) quadrant than would be expected by chance: *p* = 0.0003 for 6MO and *p* = 0.0005 for 14MO for the T quadrant and *p* = 0.0004 for 6MO and *p* = 0.0012 for 14MO for the O quadrant. The heatmaps illustrated that the spatial distributions of the track points for mice from both groups were localized to the area of the platform location (Figure 4E). The preference scores for the 6MO and 14MO groups did not differ significantly (*p* > 0.05, Mann–Whitney *t*-test) (Figure 4F). Finally, as an additional approach to evaluate the animals’ memory for the platform location, we determined the correct first choice score (CFC score) by subtracting the latency to reach the (former) platform location in the rMWM task from the latency in the sMWM task. There was no difference in the CFC scores between the groups (*p* > 0.05, Mann–Whitney *t*-test) (Figure 4G). Thus, while the spatial learning of the older mice was impaired in the rMWM task compared to the younger mice, their memory remained unaffected.

#### cdMWM

**Figure 5:**
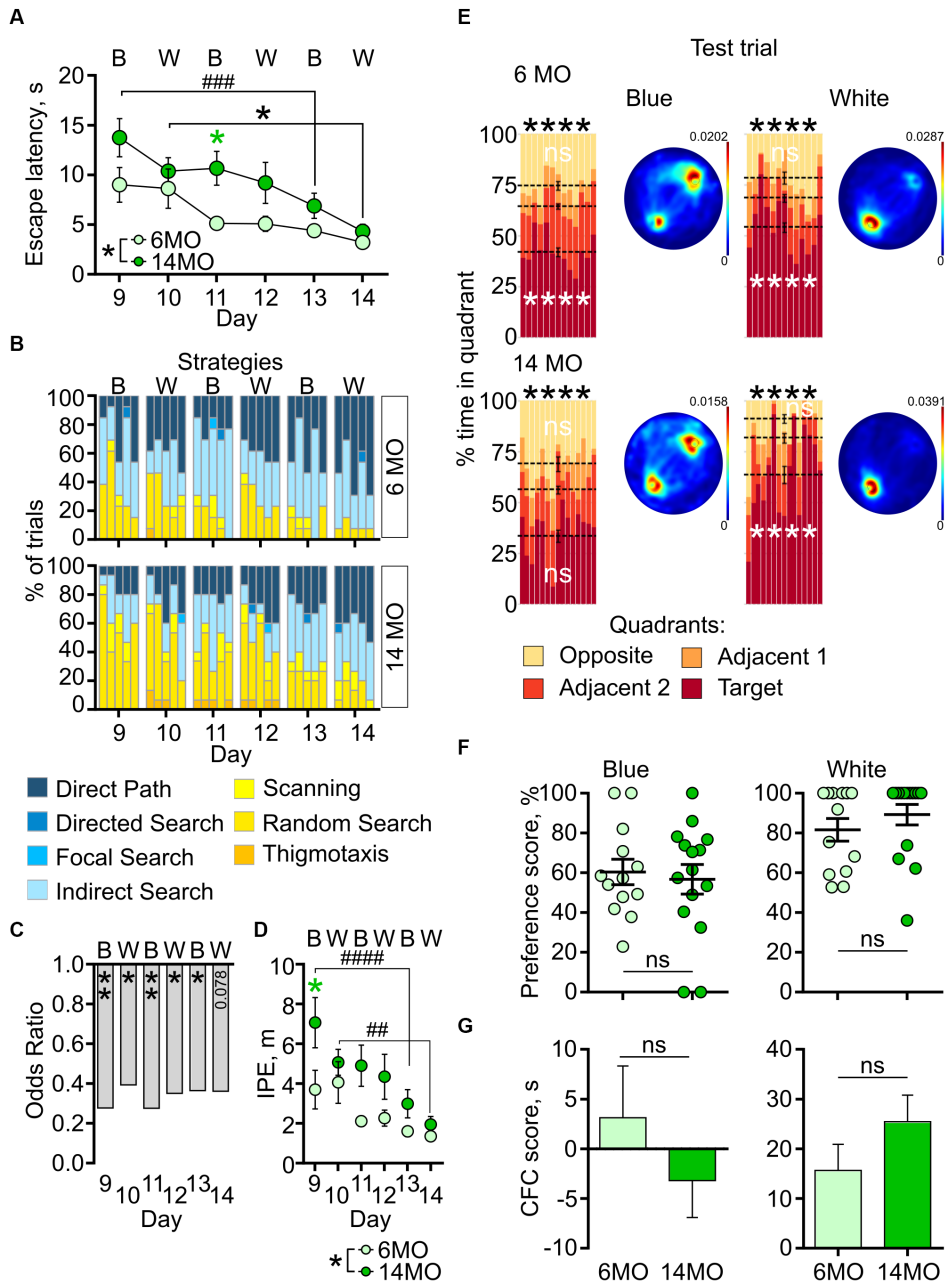
Behavior in context discrimination MWM (cdMWM) of 6-month-old (6MO, *n* = 13) and 14-month-old (14MO, n = 15) mice. **(A)** Escape latencies (time to reach a hidden platform) during training; black asterisks: both groups or effect of age, *p < 0.05, ^###^*p* < 0.001 day 9 vs day 13 for the 14MO group; green asterisk: comparison of 14MO vs 6MO on specific day, *p < 0.05 (two-way ANOVA, multiple comparisons with Sidak’s correction), data presented as Mean ± SEM; **(B)** Search strategies in cdMWM; each block of stacked bars indicates strategies used for the 5 trials for each training day; **(C)** Odds ratio of spatially precise strategies use by the 14MO group compared to the 6MO groups in cdMWM; *p < 0.05, **p < 0.01 (*p* values determined by fitting a generalized linear mixed effect model with binomial distribution); **(D)** Ideal path error (IPE) in cdMWM; black symbols: *p < 0.05 for effect of age, ^##^*p* < 0.01, ^###^*p* < 0.001 day 9 vs day 13 or day 10 vs day 14 for the 14MO group; green asterisk: 14MO vs 6MO, *p < 0.05 (two-way ANOVA, multiple comparisons with Sidak’s correction), data presented as Mean ± SEM; **(E)** Time spent in quadrants (in %) during test following sMWM training; each column represents an animal and each color represents the percent of time spent in each quadrant. Mean value for the fraction of time spent in each quadrant is represented by a black dash line and the error bar on the mean is approximated with the inverse Fisher information, ****p < 0.0001, ns – *p* > 0.05, in black symbols the results of Dirichlet distribution analysis are shown, in white symbols the results of *post-hoc* single sample *t*-test comparison (with Bonferroni correction) with the theoretical value 25% are shown; heatmaps represent average distribution of the track points (mouse locations) in the pool during test trial; **(F)** Preference score, relative time spent in T quadrant in test; dots represent individual values, ns – *p* > 0.05, data presented as Mean ± SEM; **(G)** Correct first choice (CFC) score (latency to reach the false cue – latency to reach goal cue); ns – *p* > 0.05, data presented as Mean ± SEM.

Next, we compared the performance of both age groups in cdMWM, a complex paradigm which included varying contexts and local cues, in addition to the varying platform position relative to the distal cues characteristic of the sMWM and rMWM tasks. Mice were exposed to daily alternating contexts (pool color) and different positions of the local cues [beacons (Figure 5)]. Two-way ANOVA of escape latencies revealed significant effects of the training day (*p* < 0.0001) and age (*p* = 0.0266), but no significant interaction between training day and age (*p* > 0.05). When analyzing the daily performance, *post-hoc* comparisons indicated a longer escape latency in the 14MO group than in the 6MO group on day 11 in the blue pool (*p* = 0.039) (Figure 5A). These results suggest that learning in the cdMWM task was affected in the 14MO group.

The analysis of search strategies supported the observation of impaired learning in the 14MO group. In both groups the contribution of spatially precise strategies, such as direct path, increased compared to the sMWM and rMWM tasks, starting from the first trial on day 9 (likely due to the introduction of the local cues) (Figure 5B). The reliance on spatially precise search strategies gradually increased over the training days in both groups; however, during the initial days of training, 14MO mice exhibited a noticeable delay in their adoption of these efficient strategies compared to the younger mice. Nevertheless, by the end of training, the disparity in the use of spatially precise strategies between the older and younger mice diminished.

The GLMM analysis indicated significant effects of the training day and age (*p* < 0.001 and *p* < 0.001, respectively), but not of the day × age interaction (*p* = 0.979) (Figure 5C). The ORs for using efficient strategies were significantly lower for the 14MO group compared to the 6MO group: 0.278 (*p* = 0.003) on day 9, 0.393 (*p* = 0.034) on day 10, 0.276 (*p* = 0.005) on day 11, 0.351 (*p* = 0.018) on day 12, 0.365 (*p* = 0.045) on day 13, and 0.361 (*p* = 0.078) on day 14.

Significant effects of the training day and age were also observed for the IPE (*p* < 0.0001, *p* = 0.0289, respectively), but the day × age interaction was non-significant (*p* = 0.213). *Post-hoc* analysis revealed significantly higher IPE in the 14MO group compared to the 6MO group on day 9 (*p* = 0.0228) (Figure 5D). Taken together, the results with cdMWM indicate a significant learning deficit in the 14MO mice compared to the 6MO mice.

In the memory test, similar to the sMWM and rMWM tasks, the distribution of time spent by both groups in the four quadrants of both pools significantly deviated from a uniform distribution (*p* < 0.0001 both for the 6MO and 14MO groups in the blue and in the white pool) (Figure 5E). *Post-hoc* single sample *t-tests* revealed that while the 6MO group spent significantly more time in the T quadrant than in the O quadrant of the blue pool, the fraction of time spent by the 14MO group in each quadrant did not significantly differ from the expected chance value of 25% (6MO: T quadrant: *p* < 0.0001, O quadrant: *p* = 0.7291; 14MO: T quadrant: *p* > 0.05 for both the T and O quadrants). In the white pool, both the 6MO and 14MO groups spent more time in the T quadrant than the chance value of 25% (*p* < 0.0001), while there was no significant difference in time spent by either group in the O quadrant (*p* > 0.05).

These findings were further illustrated by the heatmaps: in the blue pool, there was a higher density of track points in the area of platform, but a second focus of higher track point density was observed in the opposite quadrant; in the white pool, the density of track points was higher in the area of the platform in both groups (Figure 5E).

The preference scores and CFC scores did not significantly differ between the 6MO and 14MO groups in either pool (*p* > 0.05, Mann–Whitney *t*-test) (Figures 5F,G). To address the possibility that in the test session with the local cues present, mice might have initially visited the goal cue but, in the absence of the platform, explored other locations in the pool, and this may have blurred the difference in the preference scores, we compared the preference scores obtained during the first 10 s of the 60-s test trial in the cdMWM task; no between-group differences in preference scores were observed in either pool (*p* > 0.05). Thus, while the cumulative time spent in the T quadrant was altered in 14MO mice in the blue pool (but not in the white pool), analysis of their behavior toward the cues did not show any difference compared to the 6MO mice, suggesting that memory in 14MO mice was similar to that of 6MO mice.

In summary, compared to the younger 6MO mice, 14MO mice exhibited intact learning in the sMWM task but showed deficits in the rMWM and cdMWM tasks. Furthermore, we did not find evidence of memory impairment in 14MO mice after multiple days of training in the sMWM, rMWM, and cdMWM tasks.

### Hippocampal neurogenesis and the MWM task performance of individual mice

Certain aspects of learning and memory, particularly in complex settings, have been shown to rely on ongoing hippocampal neurogenesis. As the division of neural stem cells and production of new neurons in the DG continuously decline with age (Kuhn et al., 1996; Ben Abdallah et al., 2010; Encinas et al., 2011; Kirschen and Ge, 2019), it is plausible that age-related changes in animals’ performance in the learning and memory tests are associated with the changes in hippocampal neurogenesis. Therefore, we investigated neurogenesis in 6MO and 14MO mice and explored its potential connection to their test performance.

**Figure 6:**
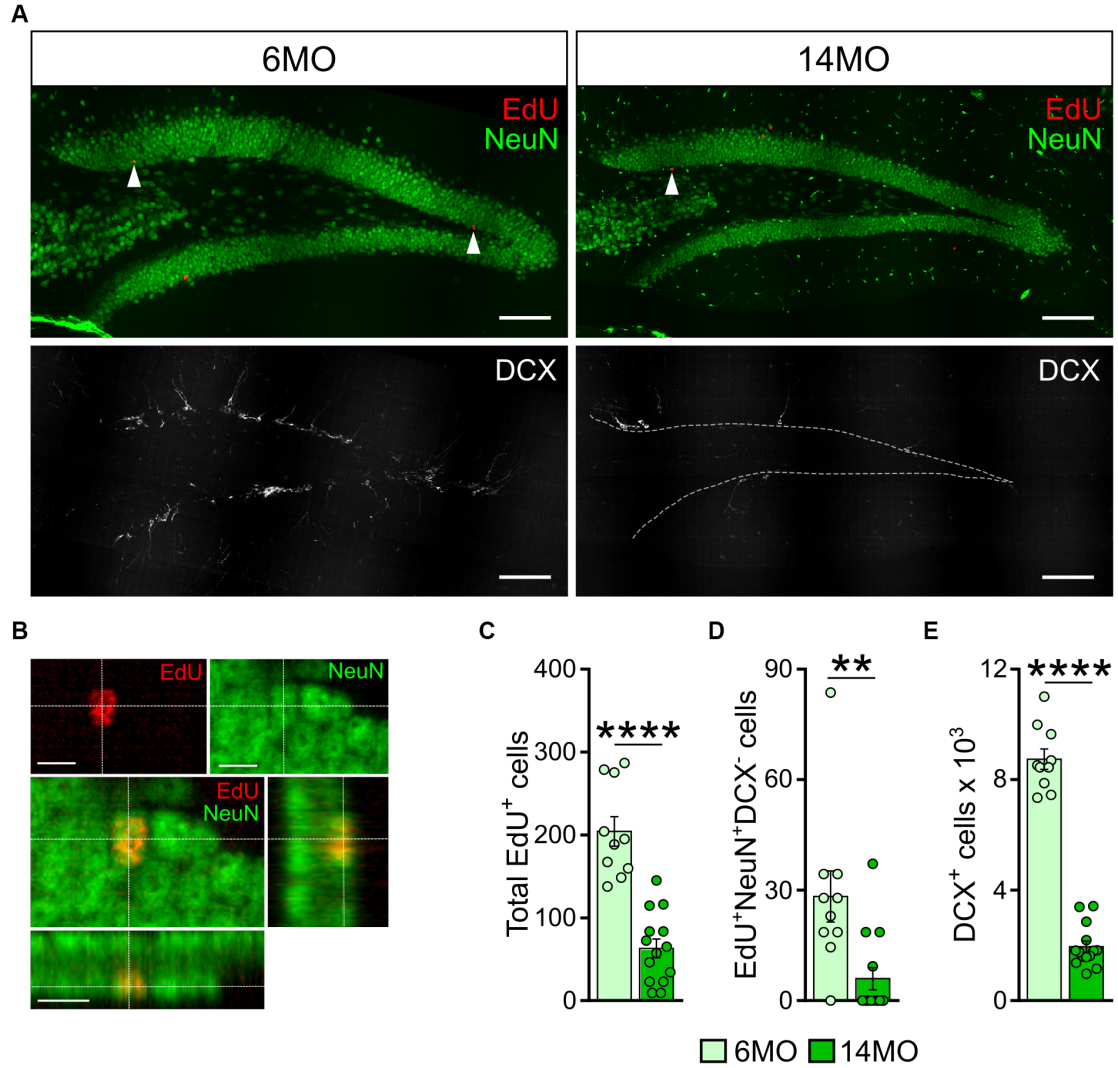
Neurogenesis in 6-and 14-month-old mice: **(A)** Images of sections from 6MO (left column) and 14MO (right column) mice stained for EdU, NeuN (top row), and DCX (bottom row); arrows on two top images indicate EdU^+^ cells; white dash line in lower right image outlines the borders of the granule cell layer of the DG; scale bar – 100 μm; **(B)** Example confocal images and cross sections representing EdU labeling co-localized with NeuN expression in the same cell of the granule cell layer of the DG of a 14MO mouse; scale bar – 10 μm; **(C)** number of EdU^+^-cells per DG; ****p < 0.0001; **(D)** Number of EdU^+^NeuN^+^DCX^−^-cells (mature adult-born neurons) per DG, **p < 0.01; **(E)** Number of DCX^+^ cells (immature adult-born neurons) per DG. ****p < 0.0001; **(C–E)** Data presented as Mean ± SEM, all comparisons – Mann–Whitney *t*-tests.

Six weeks prior to the start of learning and memory training (i.e., 8 weeks at the time of the final cdMWM test, Figure 1), mice from both age groups were administered a synthetic thymidine analog EdU. Following completion of the test, the mice hippocampi were examined for the EdU labeling and expression of NeuN and DCX markers. The EdU signal identifies cells that were undergoing DNA duplication at the time of EdU administration, NeuN expression marks differentiated neurons in the DG, and DCX expression marks advanced neuronal precursors and immature neurons (Kronenberg et al., 2003; Encinas and Enikolopov, 2008; Podgorny et al., 2018). Thus, EdU^+^NeuN^+^DCX^−^ cells correspond to newly generated fully differentiated mature neurons derived from progenitors that were dividing at the time of the EdU administration; DCX^+^ cells encompass a broad range of neuronal precursors; and EdU^+^DCX^+^ cells correspond to neuronal precursors that were in the cell cycle at the time when the label was injected (representative images are shown in Figures 6A,B).

We found a significantly lower number of EdU^+^, EdU^+^NeuN^+^DCX^−^, and DCX^+^ cells in the DG of 14MO mice compared to 6MO mice (*p* < 0.0001, *p* = 0.0018, and *p* < 0.0001, respectively) (Figures 6C–E), in line with previous reports (Bondolfi et al., 2004; van Praag et al., 2005; Gil-Mohapel et al., 2013). As expected, we did not detect any EdU^+^DCX^+^ cells in either group.

Notably, when we assessed the impact of between-sample variability on the total variability, we found that the $\frac{\mathrm{mean}\;CE_{sampling}^{2}}{CV_{group}^{2}}$ ratio was lower than 0.5 for each cell count in either group: 0.282 and 0.25 for the EdU^+^ cells in the 6MO and 14MO groups, respectively; 0.20 and 0.0133 for the EdU^+^NeuN^+^DCX^-^ cells in the 6MO and 14MO, respectively; and 0.130 and 0.0803 for the DCX^+^ cells in the 6MO and 14MO groups, respectively. This indicates that the variability introduced by the sampling procedure was less than half of the total variability, thus, supporting the validity of our cell counts (particularly relevant for the older animals).

Given the involvement of newly generated hippocampal neurons in a range of behavioral responses, we further investigated whether the number of newly produced cells in individual animals correlated with their performance in the learning and memory tasks. Specifically, we correlated the numbers of DCX^+^ and EdU^+^NeuN^+^DCX^−^ cells (representing immature and mature neurons, respectively) in each mouse with their escape latency and with their use of spatially precise strategies during the initial days of training in the sMWM, rMWM, and cdMWM tasks (day 1, day 6, and days 9–10 of the training sequence, respectively).

**Figure 7:**
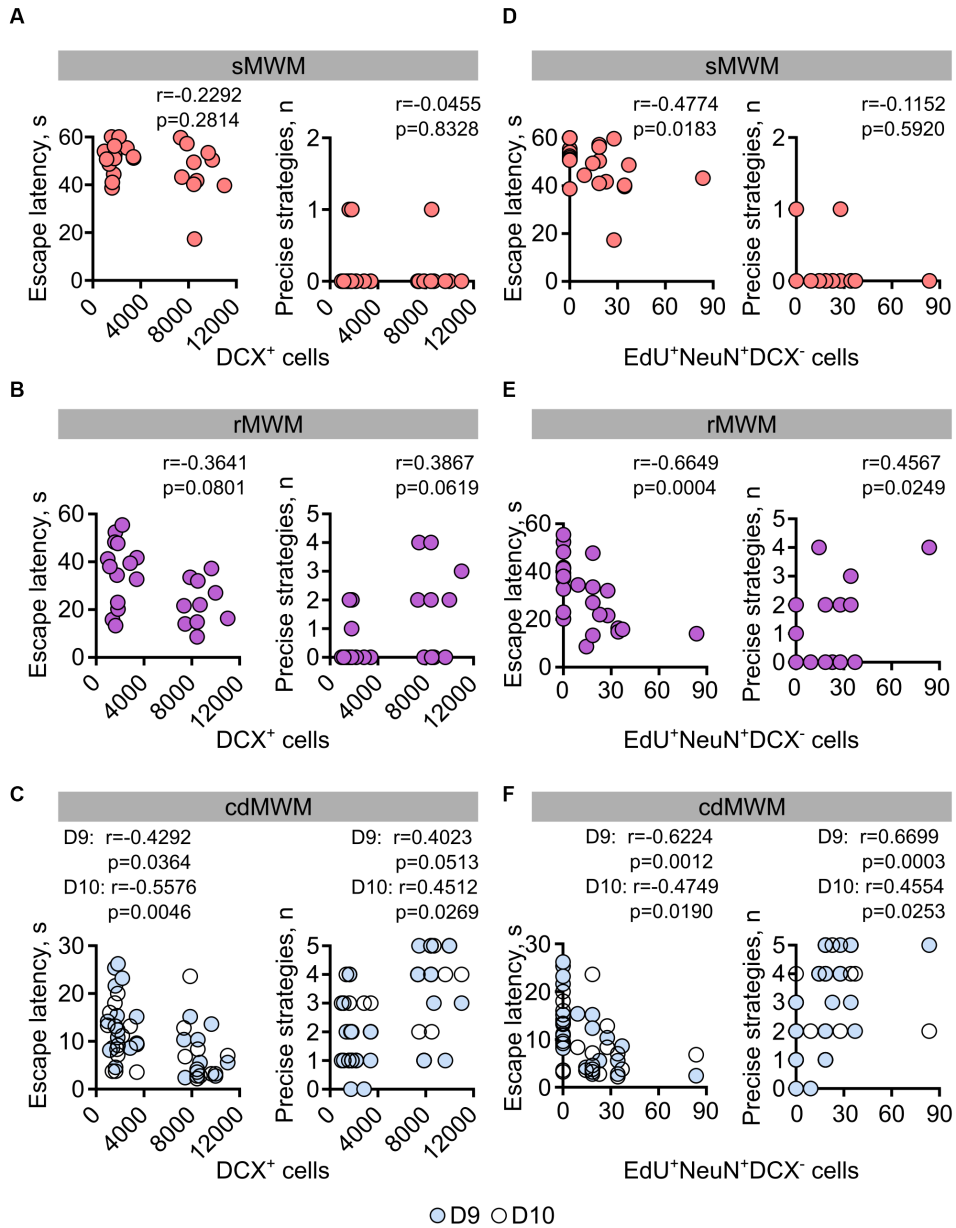
Correlation analysis (Spearman’s *r*) for mice individual performance parameters on the first days of training in: **(A,D)** – sMWM; **(B,E)** –rMWM, **(C,F)** – in cdMWM with the numbers of adult-born immature (DCX^+^) and mature (EdU^+^NeuN^+^DCX^−^) neurons.

On the first day of training in sMWM we did not find a significant correlation (Spearman’s *r* here and later) between the number of DCX^+^ immature neurons in individual animals and their task performance (Figure 7A). Of note, the evident bimodal distribution of the cell counts reflects the difference in the number of these cells between the 6MO and 14MO groups.

During the initial training in rMWM, the correlation between the number of DCX^+^ cells and the animal’s escape latency or use of spatially precise search strategies did not reach statistical significance (Figure 7B, *r* = −0.3643, *p* = 0.0801, *r* = 0.3867, *p* = 0.0619, respectively). However, on the first and second days of training in cdMWM (in the blue and white pools, respectively), there were significant correlations between the number of DCX^+^ cells and the escape latencies (Figure 7C (left graph), day 9: *r* = −0.4292; *p* = 0.0364; day 10: *r* = −0.5576; *p* = 0.0046). Additionally, there was a trend for a correlation between the number of DCX^+^ cells and the use of spatially precise search strategies on the first day of cdMWM (Figure 7C (right graph), day 9: *r* = 0.4023, *p* = 0.0513), and a significant correlation on the second day of cdMWM (Figure 7C (right graph), day 10: *r* = 0.4512, *p* = 0.0269).

On the first day of training in the sMWM, the number of adult-born differentiated neurons (EdU^+^NeuN^+^DCX^−^) was correlated with the escape latency, but not with the use of spatially precise search strategies (*r* = −0.4774, *p* = 0.0183 and *r* = −0.1152, *p* = 0.5920, respectively) (Figure 7D). The number of adult-born differentiated neurons (EdU^+^NeuN^+^DCX^−^) showed correlations with the escape latency (Figure 7E, *r* = −0.6649, *p* = 0.0004) and the use of spatially precise search strategies (*r* = 0.4567, *p* = 0.0249) on the first day of training in the rMWM task. Furthermore, the number of mature neurons correlated with the average escape latency (day 9: *r* = −0.6224, *p* = 0.0012; day 10: *r* = −0.4749, *p* = 0.0190) and the use of spatially precise search strategies (day 9: *r* = 0.6699, *p* = 0.0003; day 10: *r* = 0.4554, *p* = 0.0253) during the initial days of training in cdMWM (Figure 7F). Moreover, when comparing the data for the mice in the two age groups, we found a significant correlation (*p* = 0.0205) and a highly significant correlation (*p* < 0.0001) between the number of mature neurons and their escape latencies on day 6 of rMWM, and on days 9 and 10 of cdMWM, respectively, in individual 14MO mice but not in 6MO mice. Importantly, this correlation in 14MO mice disappeared by the end of training in each of these tasks (day 8 of rMWM, days 13 and 14 for cdMWM, all *p* > 0.05; not shown). Taken together, these findings suggest that individual animals with higher levels of adult-born immature and mature neurons perform better at the beginning of training in tasks that require flexible adaptation to the changed demands of the task.

## Discussion

Our results highlight a decline in cognitive flexibility during the adult period of mice lifespan. This decline is manifested as a reduced ability to adjust previous experiences in order to solve tasks that involve new combinations of familiar cues and contexts. Notably, the impairment of cognitive flexibility in older animals can be partially mitigated through additional training. Furthermore, our results suggest a correlation between the decline in cognitive flexibility within individual animals and a decrease in the numbers of recently generated mature neurons or immature neuronal precursors in their hippocampi. Together, our results suggest that while adult hippocampal neurogenesis may not be essential for learning a new task, it plays a critical role in circumstances that require the modification of previously acquired experience.

We focused our investigation on mature adult-middle age period of the mice lifespan (6 and 14 month at the start of the training series) to mitigate the effect of some of the confounding factors of the young and old age (e.g., sexual maturation, continuous growth, and metabolic changes in younger mice and increase in the markers of senescence and inflammation and decrease in locomotor activity and grip strength in older mice) (Flurkey et al., 2007; Yanai and Endo, 2021). Furthermore, this period is also characterized by the least relative changes in various behavioral tests related to learning and memory, anxiety, and pain sensitivity (e.g., MWM, Barnes maze, fear conditioning, open field, marble burying, shock sensitivity) (Yanai and Endo, 2021). Indeed, consistent with previous studies (Frick et al., 2000; de Fiebre et al., 2006; Driscoll et al., 2006; Gil-Mohapel et al., 2013; Shoji and Miyakawa, 2019; Yanai and Endo, 2021), we did not observe learning or memory deficits in the older group compared to the younger group in the conventional spatial version of the MWM (sMWM). We also did not detect aging effects on the repertoire of search strategies in the sMWM, furthermore, in both groups we observed a gradual increase of spatially precise search strategies during the learning trials, in line with published findings (Garthe et al., 2009; Garthe and Kempermann, 2013; Gil-Mohapel et al., 2013; Bowers et al., 2020). Moreover, the responses of both age groups were similar across all metrics that we used to assess mice behavior.

However, differences between the age groups were detected when mice were tested in the reversal version of the MWM (rMWM), which requires a degree of cognitive flexibility. Older mice exhibited longer escape latencies, a reliance on spatially imprecise search strategies, and increased IPE compared to younger mice. Interestingly, the difference between the groups was evident only at the beginning of the rMWM training, with the difference in corresponding metrics becoming non-significant by the end of training. Of note, the differences in reversal learning in the MWM have been reported for mice of contrasting ages (2 and 18 months) (Hamieh et al., 2021), but not when comparing mature adult and aged mice (Frick et al., 2000; de Fiebre et al., 2006; Shoji and Miyakawa, 2019).

The differences between older and younger mice were particularly evident in the cdMWM task (Amelchenko et al., 2023), which introduced additional variables such as alternating local cues and contexts and required further reevaluation and adjustment of the previously successful strategies. The older mice exhibited impaired performance during learning, as compared to the younger mice; this was evident across multiple metrics, including escape latency, the use of spatially imprecise search strategies during training, odds ratio of using efficient strategies, IPE, and time spent in the correct quadrant during the memory test (Figure 5). Similar to the rMWM task, the performance gap between the age groups diminished with additional training (which may explain comparable memory scores in both groups). Note that if the cdMWM had been presented as the first task, without prior exposure to the sMWM or rMWM, it would have primarily assessed the mice’s capacity for learning and memory, rather than cognitive flexibility which involves exposure to preceding rounds of learning and the ability to readjust the learned strategy in a novel setting.

Our findings demonstrate a remarkable degree of correlation between the level of hippocampal neurogenesis in individual animals and their performance in tasks that rely on cognitive flexibility (Figure 7), in line with previous reports (Drapeau et al., 2003; Leuner et al., 2004; Gil-Mohapel et al., 2013). Notably, this correlation was only partially revealed in allegedly simpler tasks that focus on efficient learning and memory, but, unlike cdMWM, do not require (sMWM) or have a lesser requirement for (rMWM) the reevaluation of previously acquired knowledge and adaption to new environment. Moreover, our results suggest that cells at different stages of neuronal differentiation play distinct roles in tasks requiring cognitive flexibility: specifically, the number of immature DCX^+^ hippocampal neurons correlated with individual animals’ performance in the cdMWM task, but not in the sMWM or rMWM tasks; in contrast, the number of mature EdU^+^ neurons correlated with performance in all three tasks.

Potentially, the increased physical activity and enriched environment during the training period and process of learning itself may affect the cascade of neuronal differentiation at various stages: stem and progenitor cell division, selective cell elimination, differentiation, and maturation of the young neurons of different ontogenic age and their integration into the preexisting circuitry (Kempermann et al., 1997; van Praag et al., 1999; Bergami et al., 2015; Denoth-Lippuner and Jessberger, 2021). In our experimental setting this might be particularly relevant to the DCX^+^ population, the bulk of which was born and has been maturing during the training period and therefore may be affected by the tasks themselves. However, the EdU^+^NeuN^+^DCX^−^ cells correspond to the cohort that was born at the time of the EdU administration, i.e., 6 weeks before the start of the training sequence, and have already differentiated and matured by the start of the training sequence; thus, their number is unlikely to be affected by the training process.

Interestingly, the correlation between neurogenesis and performance was specifically observed in the older animals. Notably, in older mice the correlation between the number of differentiated neurons and escape latency was present during the first days of training (day 6 for rMWM and days 9 and 10 for cdMWM) but disappeared by the end of training (days 8 for rMWM and days 13 and 14 for cdMWM). This may indicate that mature adult-born neurons are crucial when the animal is adjusting its relevant experience but become less critical at the later stages, when the performance of older animals approaches that of younger ones. Together, our results provide further evidence for the association between hippocampal neurogenesis and cognitive flexibility, particularly in older animals.

Our study underscores the importance of employing multiple metrics to characterize animal behavior in complex tasks, such as those assessing cognitive flexibility. Studies in which the escape latency, determined at a single time point, serves as a sole measure of the animals’ performance, may miss important differences. Dissecting the animals’ patterns of spatial navigation into defined spatially precise and spatially imprecise (and thus efficient and inefficient, respectively) strategies and following them across each trial reveals fine differences and provides critical insights into the learning process (Garthe et al., 2009; Garthe and Kempermann, 2013; Gil-Mohapel et al., 2013; Cooke et al., 2019; Berdugo-Vega et al., 2020; Bowers et al., 2020; Hernandez-Mercado and Zepeda, 2021; Yanai and Endo, 2021; Villarreal-Silva et al., 2022). Supplementing these parameters with additional metrics, such as OR, IPE, comparison of quadrant time distribution, path heatmaps, preference score, CFC score, and the correlation analysis between individual animals’ performance and their level of hippocampal neurogenesis, offers a more comprehensive and precise representation of learning, memory, and cognitive flexibility.

## Data availability statement

The raw data supporting the conclusions of this article will be made available by the authors, without undue reservation.

## Ethics statement

The animal study was approved by Institutional Animal Care and Use Committee of the Stony Brook University. The study was conducted in accordance with the local legislation and institutional requirements.

## Author contributions

EA, DB, KA, GE, and AL designed the experiments. EA, DB, OC, and AL performed experiments. GE and KA provided funding. All authors interpreted the results, made direct, and intellectual contribution to the project, and approved the final version of the manuscript.

## Funding

This study was supported by grants R01 AG057705-01 and R01 AG076937-01 to GE from the National Institute on Aging and by grant 20-15-00283 to DB and KA (learning and memory tasks in aged mice) from the Russian Science Foundation.

## Conflict of interest

The authors declare that the research was conducted in the absence of any commercial or financial relationships that could be construed as a potential conflict of interest.

## Publisher’s note

All claims expressed in this article are solely those of the authors and do not necessarily represent those of their affiliated organizations, or those of the publisher, the editors and the reviewers. Any product that may be evaluated in this article, or claim that may be made by its manufacturer, is not guaranteed or endorsed by the publisher.
